# Editorial: Cancer and nutrients: new chemicals, signals, and biomarker-based therapy

**DOI:** 10.3389/fonc.2023.1190065

**Published:** 2023-04-11

**Authors:** Xian-Yang Qin, Marco Antonio Mendoza-Parra, Yohei Shirakami

**Affiliations:** ^1^ Laboratory for Cellular Function Conversion Technology, RIKEN Center for Integrative Medical Sciences, Yokohama, Japan; ^2^ Génomique Métabolique, Genoscope, Institut François Jacob, CEA, CNRS, Université d’Évry, Université Paris-Saclay, Évry, France; ^3^ Department of Gastroenterology, Graduate School of Medicine, Gifu University, Gifu, Japan

**Keywords:** chemoprevention, nutrition, precision medicine, cancer metabolism, cancer prognosis

Nutrients such as vitamins, minerals, fats, and amino acids show promise as signaling modulators in controlling psychological and pathological processes. Altered nutrient metabolism and related signaling pathways contribute to tumorigenesis and thus serve as targets for cancer therapy. Importantly, due to their favorable safety profile, nutrients hold promise as adjunctive approaches in cancer prevention. An excellent example is the role of retinoids, a group of vitamin A derivatives, in the treatment of hepatocellular carcinoma (HCC) (1). Low serum retinol levels have been described as a risk factor for the development of HCC in humans (2), and dominant-negative mutations (3) or phosphorylation-induced inactivation (4) in retinoid receptors promote HCC development in transgenic mice. Reactivation of retinoid signaling with an acyclic retinoid, peretinoin, showed promising therapeutic results in clinical trials to prevent the recurrence of HCC after curative treatment (5, 6).

However, the clinical application of nutrients is still challenging due to the ambiguous evidence of effectiveness and lack of mechanism-based biomarkers to identify a high-risk subgroup. The aim of this Research Topic on “Cancer and Nutrients: New Chemicals, Signals, and Biomarker-Based Therapy” is to establish the current views of the chemical and biological basis of natural and synthetic nutrients in cancer biology, such as tumor immunology and microenvironment, and to collect evidence of nutrients in cancer epidemiology and clinical practice.

## Chemical and biological basis of natural and synthetic nutrients in cancer biology

Metabolic reprogramming is a well-recognized cancer hallmark that contributes to the biosynthesis of energy and nutrients for cancer cell processes, as well as the regulation of oncogenic signaling pathways (7). Tsuchiya focused on the role of hepatic iron overload (HIO) in the pathology of chronic liver disease and HCC, and comprehensively reviewed current evidence of nutritional interventions, including vitamins (A, C, D, E), adenine, zinc, niacin, folate, and riboflavin, in targeting iron metabolism for HCC prevention. Particularly, the author discussed liver fibrosis as a key factor in HIO-induced hepatocarcinogenesis and proposed hepcidin-mediated systemic iron metabolism and HIO-induced ferroptosis as potential therapeutic targets for future studies. Zhang et al. focused on nitrogen metabolism disorder (NMD) in the occurrence and development of lung adenocarcinoma (LUAD). The authors proposed a novel idea that high nitrogen metabolism levels are associated with poor LUAD prognosis. This is not only because nitrogen is a main source for the synthesis of nucleotides and proteins in cancer cells, but also because it acts as a negative regulator of activated dendritic cells and Type II interferon response in immune cells, thereby affecting immune function. Philips et al. focused on the bioactive functions of endogenous metabolites in post-translational modifications (PTMs), including lactylation, serotonylation, and succinylation, of proteins, especially histones, in cancer progression. The authors also discussed newly discovered functional metabolites with unknown modes of action and recent advances in mass spectrometry and bioinformatics technologies to identify new bioactive metabolites and examine protein-metabolite interactions. It is convincing that targeting metabolite-based PTMs is an exciting new field for cancer prevention and treatment. Regarding PTMs, Liu et al. focused on ubiquitination and deubiquitination and reported the oncogenic role of the ubiquitination regulator, MARCH9, in colorectal cancer (CRC) progression. Knockdown of MARCH9 inhibited, while MARCH9 overexpression promoted CRC cell proliferation and migration, partly by downregulating the expression of a deubiquitinase cylindromatosis gene and activating p65, a member of the nuclear factor-κB protein family. Therefore, MARCH9 may be a novel and effective therapeutic target for CRC therapy.

## Epidemiological evidence of nutrients in cancer risk and prognosis

The dose-response relationship is a crucial indicator for cancer risk characterization. Zhao et al. conducted a systematic review and quality appraisal of clinical practice guidelines (CPGs) related to nutrition management (NM) for patients with head and neck cancer (HNC) during the peri-radiotherapy. The authors noted discrepant recommendations and the absence of essential parts of existing CPGs based on the AGREE II Instrument. They comprehensively summarized the recommendations across all guidelines for NM, which provides an excellent collection of current protocols and a critical reference for clinical practices of NM in HNC therapy. Luo et al. focused on the dietary inflammatory index (DII), which quantitatively assesses inflammatory potential of the overall diet using food-frequency questionnaires. The authors conducted a comprehensive and critical systematic review to address the dose-response relationships between DII scores and oral cancer risk. They found that the risk of oral cancer increased by 135% at the highest DII level compared to the lowest DII level, which was influenced by adjustments for socio-economic status, rather than family history of cancer. This study supports the notion that DII may serve as a monitoring indicator to optimize dietary guidelines and as a predictive biomarker to identify a high-risk subgroup in the prevention of oral cancer. Gu et al. focused on the risk of environmental and dietary exposure to acrylamide (AA) on cancer mortality using data from the National Health and Nutrition Examination Survey (NHANES) study. The population-based retrospective analysis showed a dose-dependent increase in cancer mortality with levels of serum hemoglobin adducts of AA, HbGA, and HbAA, and proposed that the low-grade inflammation score played a mediated role in this process. This study provided direct evidence for the effects of AA exposure on cancer mortality in the general population and highlighted the importance to restrict or control public exposure to AA from various sources, such as smoking or AA-rich foods.

Overall, this collection makes a significant contribution to the literature by clarifying the efficacy of nutrients and informing further efforts in promoting clinical research into nutrients in cancer prevention and therapy from the perspective of biomarker-based precision medicine.

## Author contributions

All authors listed have made a substantial, direct, and intellectual contribution to the work and approved it for publication.
